# The Role of Soundscape in Nature-Based Rehabilitation: A Patient Perspective

**DOI:** 10.3390/ijerph13121229

**Published:** 2016-12-11

**Authors:** Gunnar Cerwén, Eja Pedersen, Anna María Pálsdóttir

**Affiliations:** 1 Department of Landscape Architecture, Planning and Management, Swedish University of Agricultural Sciences, SLU, SE-230 53 Alnarp, Sweden; 2 Environmental Psychology, Department of Architecture and Built Environment, LTH, Lund University, SE-221 00 Lund, Sweden; eja.pedersen@arkitektur.lth.se; 3 Department of Work Science, Business Economics and Environmental Psychology, Swedish University of Agricultural Sciences, SLU, SE-230 53 Alnarp, Sweden; anna.maria.palsdottir@slu.se

**Keywords:** garden therapy, soundscape, design, health, mental restoration, nature-based rehabilitation, soft fascination, horticulture therapy, therapeutic landscape

## Abstract

Nature-based rehabilitation (NBR) has convincing support in research, yet the underlying mechanisms are not fully understood. The present study sought to increase understanding of the role of soundscapes in NBR, an aspect paid little attention thus far. Transcribed interviews with 59 patients suffering from stress-related mental disorders and undergoing a 12-week therapy programme in the rehabilitation garden in Alnarp, Sweden, were analysed using Interpretative Phenomenology Analysis (IPA). Described sounds were categorised as natural, technological or human. The results showed that patients frequently referred to natural sounds as being part of a pleasant and “quiet” experience that supported recovery and induced “soft fascination”. Technological sounds were experienced as disturbing, while perception of human sounds varied depending on loudness and the social context. The study further uncovered how sound influenced patients’ behaviour and experiences in the garden, through examination of three cross-theme dimensions that materialised in the study; sound in relation to overall perception, sound in relation to garden usage, and increased susceptibility to sound. The findings are discussed in relation to NBR; the need for a more nuanced understanding of susceptibility to sound among people suffering from mental fatigue was identified and design considerations for future rehabilitation gardens were formulated.

## 1. Introduction

### 1.1. Soundscape Research

Research on soundscapes—as in the study of the experience of the acoustic environment [1,2]—was first initiated in the late 1960s [3,4]. In recent years, there have been substantial developments in the field [5,6]. There is now an increased understanding of the contextual experience of sound, and also of how the sonic environment can be influenced in a strategic manner to improve the overall soundscape. Previously, such environmental considerations had been limited to a “defensive” approach [7,8]. In a defensive strategy, the main intention is to protect human beings from unwanted sounds (noise). However, while the defensive strategy has been a dominant and useful approach in environmental planning for many years, it has failed to pay attention to the actual experience of sound. In the opposite and more positive approach to sound, sometimes referred to as “offensive”, the focus shifts from noise to consideration of what people want to hear. These approaches, together with a third (“creative” approach), may be used interchangeably in order to improve the sonic environment [9]. In such a holistic soundscape approach, consideration of unwanted sounds (noise abatement) is combined with consideration of wanted sounds for improved experience and/or masking effects [10]. For instance, soundscapes can be influenced through noise screening [11], localisation of functions [12], creation of biotopes for birds [13], consideration of walking material [14], introduction of water features [15,16], rustling vegetation [17] or sound art [8,18].

The relationship between sound and mental health has mainly been studied from the perspective of the adverse effects of noise on humans, either directly or through stress reactions induced by negative associations [19,20,21]. There are clear indications of an enhanced risk of impaired mental health after long-term noise exposure. It has been suggested that greenery could mitigate the negative impact, so that the sound is perceived as less annoying if green urban areas are provided. Several studies have found that this is the case sometimes, but not always [22,23]. The provision of sound in green environments intended for psycho-physiological restoration purposes and whether this has an impact on the link between the physical environment and possible curative effects is less well studied, but an increasing number of recent studies point to such connections. In an investigation in which subjects were exposed to a psychological stressor [24], it was shown that stress recovery was faster during exposure to nature sounds at 50 dBA rather than to three different types of noises (40–80 dBA). Similar findings were reported in a study [25] where subjects exposed to virtual reality nature (including sound) recovered faster after stress than subjects exposed to virtual reality without sound. It has also been shown that sounds perceived as pleasant (most typically birdsong, music and ocean sounds) can reduce skin conductance level for subjects at rest [26]. In healthcare, studies have shown that sounds of nature from speakers can have positive effects during difficult procedures, reducing stress and anxiety [27,28] as well as experienced pain [29].

In order to understand the experiential dimension of sound, it is necessary to go beyond measurements of sound pressure levels [9]. Soundscape research provides a platform for discussing perception of sound in terms other than annoyance, as well as opening up new possibilities for design and management of sounds. A soundscape includes all types of sounds in an environment, with emphasis on how they are experienced in a context [1,5,6].

### 1.2. Nature-Based Rehabilitation and Stress

Problems relating to mental health are estimated to be among major reasons for work disabilities globally [30,31], and can have severe negative effects on everyday lifestyle [32] and in the long run lead to physical and mental depletion [33]. There have been reports that nature and nature-like environments can assist in mental recovery [34]. It has also been shown that by spending time in natural environments, concentration ability and directed attention can be improved [35,36], and perceived stress relieved [37].

In nature-based rehabilitation (NBR), it has been found that sensory stimuli of outdoor nature experiences can play an important role in treatment of stress-related mental conditions such as exhaustion disorder [35,38,39,40]. Caregivers in NBR claim that the connection to nature through sensory impressions can help patients “open up” to treatment [38]. The role of sensory experience in NBR could possibly also be related to the notion of ‘soft fascination’ in attention restoration theory (ART) [41], an important form of experience that is useful for mental recovery. According to ART, soft fascination occurs when people experience things without a focus or specific demands. This recovery allegedly occurs in nature or nature-like environments, where the subject is free and able to discover, recover and relax.

A less well studied sensory input in the NBR context is sound, although this was indicated to be a potentially important aspect in a semi-structured interview study conducted previously with 59 former participants at Alnarp Rehabilitation Garden, where the role of nature as a supportive environment was explored [42]. A new aspect of NBR for individuals recovering from stress-related mental disorders was identified, i.e., social quietness, referring to the need for solitary encounters with nature without disturbance by others. Perception of sound in the rehabilitation garden was not a specific topic in that study, but the participants mentioned sound as an important hindering or supportive factor in a therapeutic environment. This finding suggested the need for a follow up analysis, in which sound was given more thorough attention.

The aim of the present study, consequently, is to examine the role of sound in NBR for individuals with stress-related mental disorders and to identify essential aspects for the future design of restorative spaces for mental recovery.

## 2. Methods

The study was based on 59 semi-structured interviews with individuals recovering from stress-related mental disorders after 12 weeks of NBR. The interviews were initially part of a previous study focusing on the role of nature in the NBR context [42]. In the present study, the same material was re-analysed, but with the focus on sound. The data collection procedure is summarised in the following paragraphs (for more details see [42,43]). The specific analysis procedure used in the present study is then described.

### 2.1. The Informants

All informants (*n* = 59) had participated in a 12-week NBR programme at the Alnarp Rehabilitation Garden (Lomma, Sweden). The inclusion criterion for participating in the programme was one of the following International Classification of Disease (ICD-10) codes as the primary diagnosis: psychiatric diagnosis of adjustment disorder and reaction to severe stress (ICD-F43), such as exhaustion disorder (ICD-F43.8a) or depression (ICD-F32.0 and F32.1). The definition of exhaustion disorder includes, amongst other impairments, sensitivity to sound [33,44]. The informants, 50 females and nine males, were all Swedish citizens and varied in age between 25–62 years. The relatively high proportion of female informants (85%) can to a great extent be explained by an overrepresentation of females among people that are sick-listed for stress-related mental disorders in Sweden [45].

### 2.2. The Venue and Nature-Based Rehabilitation Programme

**Figure 1:**
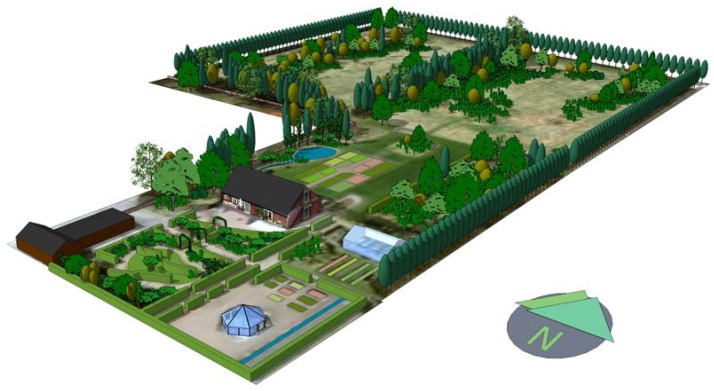
3D sketch depicting the Alnarp rehabilitation garden. Image rendered by: Gunnar Cerwén.

The NBR took place in a designed garden (Figure 1 and [46,47,48]) located at the campus of the Swedish University of Agricultural Sciences in Alnarp. The garden contains rooms for rest and recovery as well as work and social interaction. The 2-ha garden is divided into two main areas, a nature-like area in the north and an area for cultivation and gardening in the south.

**Figure 2:**
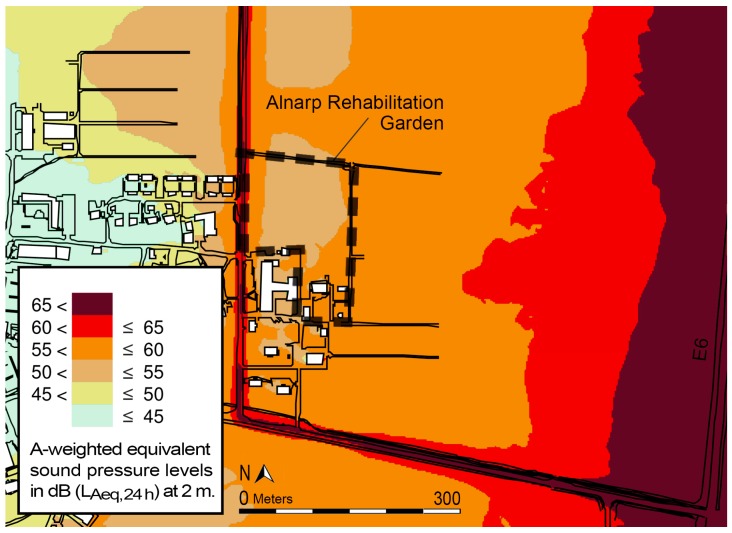
Noise map, illustrating A-weighted equivalent sound pressure levels in dB (L_Aeq, 24 h_), in and around the garden. Calculations are for 2 m above ground level and include all state- and municipal roads. The map was extracted from a noise survey commissioned by the Lomma municipality [50].

The garden design includes health-promoting characteristics such as sensory experience through sight, smell, taste and sound [46]. For instance, the wild area contains rustling trees and fruits, while there is a small waterfall in the south-east end of the garden and a pond with a small stream near the entrance. A motorway (E6) runs around 0.6 km east of the garden, exposing it to some traffic noise, especially in the south-eastern parts (Figure 2). The NBR programme was carried out as group therapy with eight participants each, supported by a multimodal rehabilitation team. The programme was provided four hours a day, four days per week, throughout its 12-week duration [43], thus ensuring frequency and length of nature exposure [49].

### 2.3. Data Collection

The interviews were conducted during 2007–2012, after the 12 weeks of NBR ended. All subjects who participated in the programme (*n* = 103) were invited for a follow-up interview. Altogether, 59 individuals accepted the invitation and participated in the study. The subjects that accepted the invitation were representative for the group as a whole in terms of age and gender. The interviews were conducted with an informal approach that was aided through incorporation of an interview guide that focused on the patient’s experience of the rehabilitation and the natural environments in the garden. Each interview lasted about one hour and was recorded and verbally transcribed. The interviews were conducted over a period of five years covering all seasons, i.e., spring, summer, autumn and winter. As recommended by the Board of Science Ethics, Lund, all participants were asked to give their written consent before voluntarily entering the study.

### 2.4. Data Analysis

A systematic search was conducted through all the transcribed interviews in order to extract information on perceived sound and soundscape. Altogether, 14 keywords describing sounds (Appendix A, Table A1) were used to search through the interview material. Each interview was treated individually. If a keyword was found in the interview, the whole section in which sound was discussed was cut and pasted in one document.

Once the search was completed, each of the three authors separately analysed the collected interview text in four steps, using Interpretative Phenomenological Analysis (IPA) [51] as a framework.

- Step 1: Categorisation of the sounds mentioned into natural, technological and human sounds [52,53] (hereafter denoted ‘sonic themes’).
- Step 2: Identification of cross-theme dimensions emerging from the text.
- Step 3: Agreement on identified themes and categorisations, reached through discussions among the three researchers based on their individual findings.
- Step 4: In-depth analysis allocating meaning to the themes.

## 3. Results

The systematic search resulted in a text document consisting of around 9000 words and including descriptions of sound from 27 of the 59 participants. The descriptions referred mainly to sounds in the rehabilitation garden, but also included recollections of other contexts and environments. The descriptions were approximately evenly distributed among the three sonic themes; natural, technological and human sounds. The findings in each of these themes constitute the first set of results (Section 3.1), while the second Section (3.2) deals with the cross-theme dimensions that emerged in the analysis.

### 3.1. Sonic Themes

#### 3.1.1. Natural Sounds

Stories about natural sounds in the rehabilitation garden were generally positive and were dominated by wind-induced sounds from vegetation and the sound of running water and of singing birds. Participants tended to describe natural sounds in a detailed and rich manner, often emphasising their own positive experience and participation in the garden. Natural sounds were further often discussed in *relation to other sensations*, such as smell, touch, visual impressions and colours. Several of these descriptions seemed to indicate a relationship to soft fascination. The following is an example from one of the participants who was asked to describe a favourite place in the garden:

“I chose it because of the bamboo behind it, where you… where I sat so I could see the pond and so. And bamboo rustles, well first of all it is green and then there is a sound in it all the time which I like, this thing... yes. It moves in the wind and there is a sound... and then there was… and looking at the water, I like that, and I think there were even little frogs in the water.”

When participants talked about natural qualities, they commonly also spoke about *silence*. Words such as silent, calm and quiet were frequently combined with nature and other words to describe a positive state, often emphasising a relative state (most typically in relation to urban environments). In its strictest of meaning, the way participants used descriptions of silence was contradictory. For instance, it was common for participants to use silence in the same sentence as descriptions of sonic events, though only natural sounds were described in this manner:

“But you know that feeling of it just being you and nature and it’s completely quiet, what, you hear a stream trickle somewhere and you hear a bird that… you hear some slight rustling in the trees or whatever, just wonderful and it’s so incredibly beautiful that you just…”

Furthermore, sounds of nature experienced in the rehabilitation garden seemed to be able to *awaken participants’ memories* concerning e.g., activities they had undertaken in the past, or connections with loved ones:

“The feature I seem to recall most clearly are the birds. I mean, I have always enjoyed bird sounds and the same thing goes for the beach, the seagulls like when I lie out on the shore and out there in a house that, instead of calling a hovel, I have chosen to call a chateau.”

#### 3.1.2. Technological Sounds

Technological sounds were mainly described from other experiences than that of the rehabilitation garden and were perceived as negative and annoying. Most commonly described was noise from road traffic in and around the participants’ homes and the feeling of wanting to *escape* from that, but sounds from e.g., computers and fans were also mentioned. The possibility to get away from this, and the perceived relative silence of the garden, was in this sense positive. One participant described it thus:

“It’s all that noise from cars and… town and... this is getting away from that, getting out, and then I think too that you discover your creativity again, that you kind of want to create, it’s the kind of environment where you feel more enthusiasm for doing things.”

In the rehabilitation garden context, most participants described the garden as quiet, although several also mentioned the problems they had with the noise from the nearby motorway, which was defined as annoying. Whether or not the motorway was annoying seemed to be related to the participant’s *life situation*. For some, the rehabilitation garden was a place without the annoying traffic noise they experienced at home, while for others—living in a quiet environment—the noise from the motorway became intrusive:

“We had an exercise here where we had to go out and sit down and just be quiet and listen. And then we had to draw, a doodle or whatever we felt like. But I wasn’t able to do that because I could only hear cars, lorries. I heard an ambulance, I heard… and I don’t get that at home. At home it’s quiet.”

#### 3.1.3. Human Sounds

Human sounds, the third and last of the sonic themes investigated, received the most varied responses. The human sounds discussed were mainly sounds in the rehabilitation garden, such as people talking to each other or moving around. Several participants described how they were distressed by social situations, some even by the sound from the therapist giving instructions. This annoyance seems to have been worst in the beginning of the therapy.

However, human sounds were in some situations also perceived as positive, contributing to the participants feeling safe. The presence of the therapists in particular seemed to have had a positive effect in this way. The sound of the therapists’ voices was described as soothing by several participants. For instance, when asked to describe what it was that made the garden seem tranquil to them, one participant said:

“I don’t know actually. I think it has a lot to do with... with you could say the way Lena and the others spoke, they talk quietly and are very careful to say that...”

The therapists spoke deliberately with calm and slow voices, and this seems to have had a positive effect on participants. Tempo, loudness and other *physical qualities of spoken words* can be important cues in perception of human sounds. This tendency was noted, not only regarding the therapists’ voices, but also in other social situations. One participant, for instance, described how a loud conversation interrupted the treatment:

“And we were just sitting there in the “Growpoint” in a chair and really enjoying it and then one of the other people in the group felt bad and stood like and talked loudly with Lotta about in there, what. Well, then I was, I actually couldn’t really bear to listen, I got kind of unwillingly… involved in it and I got so fed up of that so I was really insensitive and I said something like suddenly when I couldn’t stand to hear it.”

*Having control* over the situation and avoiding risking unwanted encounters with others were expressed as important. The garden was designed so that the participants could choose to place themselves at different degrees of physical proximity, giving them opportunities to look out on the garden, choose whether to be with other visitors, or not. A social code based on mutual respect seemed to have developed subconsciously, so that certain places in the garden were considered social and other places private. The code also included reading other participants’ body language. Sound played a role in this avoidance or interaction with others; one participant described, for instance, how the sound of footsteps on the gravel-covered walkways gave a signal that someone was approaching, thus providing a constant warning of potential participation in social interaction.

“You can hear someone coming too, since there are these gravel paths in between. [...] That’s probably mainly the reason why I did not choose [to sit at] the rear side, because it has no gravel paths. So there you have no warning, I could just be sitting there and someone could come round the corner…”

### 3.2. Cross-Theme Dimensions

#### 3.2.1. Sound in Relation to Overall Perception

As already noted in Section 3.1.1, the participants frequently associated auditory experiences with visual impressions. Natural elements in the garden, such as trees and bushes, were thus typically described with reference to two or more senses that interacted. This interplay between senses seemed to enforce a positive overall impression:

“And then outside these, since there were no walls and that, there you could see how they [the trees] swayed a little and hear a slight rustle of the leaves, and it was a really lovely place that place.”

However, man-made features could also be experienced in a similar manner. Some participants described the special, soft sound produced when walking on wood as a positive experience. The sound of wood when walked upon could be construed here as a connection between the participant and the garden, reinforcing and confirming their own movement and presence in that space. Descriptions relating to their own movement were—in a similar manner as natural sounds—often associated with different sensory experiences. There were other descriptions of walking on material than wood, but the sound of the wooden plates in the garden seems to have been especially pleasant, as it produced a soft sound reminiscent to a pier in the water.

“Then I think it’s been lovely to walk because here it’s wood... the wooden plates. They have a special sound, exactly. Very special, a slightly soft sound, while they also remind you of walking on a jetty, it’s a bit of that sound too.”

Furthermore, the differences in quality between different walking materials in the garden seem to have had an effect on participants’ speed of movement, so that they slowed down after having walked on (hard) gravel and into softer material such as the wooden plates or wooden chips.

The possibility for participants to interact with the garden on their own terms seemed to be an important aspect. Yet this patient-garden interaction could be interrupted by noise, or sounds from other participants in the garden. Such interruptions moderated the experience in a negative way, and could make it difficult to take in experiences of the garden. One participant, for example, described how it was difficult to appreciate the presence of nature while in the company of others:

“Yeah, since then it’s more like you can take in your experience more than when you’re sitting there and talking to someone or I just wanted to be by myself and just be able to relax in it, kind of…”

Similarly, technological sounds could hinder the interaction with the garden and hence have an effect on the therapy. The sound from the motorway—in addition to being an intruding feature in itself as discussed in Section 3.1.2—could also make it difficult to hear the sounds of nature. This problem was mentioned by one participant who noted how the sound of the motorway seemed to mask out the sound of birds, so that they were not as audible as they would have been if the motorway had not been there. However, masking effects in the garden could also work in the reverse way, i.e., that sounds of nature could mitigate the negative impact of noise. The same participant described how when situated by the rippling water of the pond, the sound of the motorway seemed to disappear:

“Yes, I could stand and see the ripples and listen, it was able to take away the lorries... well the traffic... it took that away. And I listened to the rippling...”

#### 3.2.2. Sound in Relation to Garden Usage

In several cases, it was clear that participants preferred some locations within the rehabilitation garden to others, based on sonic characteristics of these environments. Such choices could be made in order to avoid certain sounds (most typically human sounds, but also technological), or to embrace certain sounds (most typically nature sounds). The following example illustrates how the sound from the rippling pond in the garden was embraced and contributed to one patient’s experience and choice of place during an exercise that was part of the rehabilitation programme.

“Yes... I was sitting there once. One time we were given an exercise to go out and sit in the garden, on just one occasion. And I sat here beside the pond, or whatever you call it, that, and there was such a lovely sound of running water, so I actually just sat there, one time I sat there.”

Another participant, who was disturbed by the noise from the motorway, described how a deliberate choice to be in a part of the garden that was exposed to as little noise as possible was made. The sound of the motorway thus influenced their behaviour in the opposite way to that described in the previous example.

“Mm and then I was here too because I was really, really disturbed by this. I am quite sensitive to sounds and this motorway noise, it was really stressful for me. And you could hear it least over here. Since I grew up in the country and in silence in a way, so for me that motorway noise, you can’t shut it out and it’s just a constant stressful sound that...”

Some areas of the garden were perceived as socially quiet zones, even though there were no signs or acoustic arrangement to keep these zones quiet. Where and how participants placed themselves constituted a signal to the others on whether they sought quietness or were open for social interaction. One participant described how the smoking area (even though it had a central position in the garden) worked as a refuge where social interaction was not possible.

“Yeah, but you still have a bit of an idea about what’s going on. But nobody comes up, there was another girl who smoked and often, we never went and smoked together but nobody goes up to smokers... You don’t do it. No, no you went and smoked, kept away, so it’s like perfect, because I was like... oasis and solitude as a smoker.”

Whereas the smoker’s lounge was coded as private, other places were coded as social where the presence of others was accepted, or even appreciated. When describing the need for solitude, one participant reflected on how different places seemed to have different social meaning encoded in them:

“And this is also a social place. There were many who walked and gardened there… and it felt like people didn’t talk. When you went here there was not as much socialising in the same way as there was here, or here...”

#### 3.2.3. Increased Susceptibility to Sound

Among the participants there were some who spontaneously mentioned that they had not been sensitive to noise before they got ill and those who classified themselves as generally noise sensitive, including one participant who suffered from tinnitus towards ambient sounds. Regardless of sensitivity history, several of the participants had experienced that they became more susceptible to sound when they became ill. This transition then happened rather suddenly.

The urge to get away from the sounds of the city, i.e., technological and human sounds, could in several cases be associated with an oversensitivity to sound. However, in the worst stages of the illness, the sensitivity to sound could also include natural sounds. One participant recalled how, before the treatment, it was difficult to cope with birdsong or even small sounds from water dripping outside the window. After spending time in the rehabilitation garden, the ability to enjoy natural sounds such as birdsong was regained. Some participants stated that they re-learned to listen. The rehabilitation garden and activities within provided a possibility to enter a stage in which it was possible to tolerate, and even appreciate, the sound of nature again.

“When I started feeling burnt out I found […] it was never quiet, all the traffic and all the voices and all the neighbours. Suddenly I became oversensitive to all sound […] I couldn’t bear many sounds at all. [...] No, not even the birds. So all that has come back through this learning to relax again. [...] You forget how to do it.”

In their previous stressful life, participants had not been aware of sounds around them, but the NBR brought with it an increased sensitivity to sounds. However, the enhanced listening that was facilitated in the rehabilitation garden did not only lead to positive experiences. For one participant, it meant an increased annoyance with noise in the home environment.

“And now I’ve got so extremely sensitive to sound. In my flat I can hear sounds I never thought about before […] I have just complained to the warden about the neighbour above […] It’s just become a whole new level of noise, sort of.”

## 4. Discussion

### 4.1. Role of Sound in Nature-Based Rehabilitation

Most typically when discussing sound and health, the concern is with disturbance from unwanted sounds, i.e., noise. The present study confirmed the importance to avoid unwanted sounds, such as noise from infrastructure and/or intrusive human sounds in the context of NBR. The findings further indicated that such disturbance can be relative and dependent on experiences from other environments and/or life situations. It was also found that patients frequently referred to “quietness” as an ideal state to aid the recovery process. These references generally excluded human and technological sounds—but often included natural sounds.

The study illustrated some of the ways in which positive experience of natural sound can play a role in NBR, helping subjects recovering from stress-related mental disorders. Sounds of nature were generally given rich and colourful descriptions that incorporated the patients’ own experiences; the nature sounds were prone to evoke memories and were often described together with other sensory stimuli. In many cases, descriptions of nature sounds indicated that they (especially together with other sensory input) could induce soft fascination [41] and result in possible mental restoration. This finding thus suggests that the interaction between different sensory inputs could be an important aspect to consider. In a previous comparative study of restorative effects between nature and a simulated (visual) natural environment [54], it was found that the group exposed to real nature experienced increased energy and altered states of consciousness compared with the group exposed to virtual nature. One difference between the groups that materialised was the relative sensory connection.

In addition to natural sounds, sounds of movement emerged as positive in the study. When patients moved in the garden, the sound of their own footsteps on different materials confirmed the movement and generated a positive effect. This role of sound in the environmental experience is interactive, because of the patient’s own involvement in the creation of the sound. The interactive potential of sound has been noted previously [55] and could be extended to include effects such as echo and spatial reverberation. In our study, feedback from the different materials that were walked upon seemed to influence the pace with which patients’ walked. The potential of soundscape to influence walking pace has been reported previously [56] and it has also been suggested that there is a relationship between slow walking pace and reduced arousal [56,57].

Our study was based on the same interview material as a previous study [42], in which the participants’ need for social quietness when engaging alone with nature was highlighted. Human sounds and social contexts were described as disturbing and the need for solitude was profound. It was shown that, especially in the early phases of the treatment, the patients’ state of mind could not cope with such demanding stimuli. The present study showed that sensibility to social quietness—in addition to treatment phase and individual differences—could depend partly on physical characteristics of human sounds. Loud human sounds, for instance, were perceived as particularly disturbing, while the soft and gentle sound of the therapists’ voices could have an opposite, positive effect on rehabilitation.

In combination, the presence of natural sounds (e.g., [24,25]) along with sounds from walking materials [14,56], social quietness [42] and absence of technological sounds [19,58] could be regarded as important prerequisites for mental restoration and recovery from stress, and therefore considered essential aspects for designing restorative places.

### 4.2. Increased Susceptibility

Increased susceptibility to sound was described by the participants in connection with their illness. Their stories comprised an array of different states, including being sensitive to technological and/or human sounds, not being able to bear any kind of sound, or being unable to listen. Susceptibility to sound is mentioned in the list of criteria for the diagnosis of exhaustion disorder set up by the National Board of Health and Welfare in Sweden [44], which mentions increased sensitivity to sound among physical symptoms such as aches and pains, palpitations, gastrointestinal problems and vertigo, but does not define it. There is a need to better understand the increased susceptibility among people with exhaustion disorder, so that soundscapes supporting recovery in different stages of the illness could be included in therapeutic environments.

There are basically two established categories of increased susceptibility, namely noise sensitivity and hyperacusis (though several other terms are used). The main distinction is that noise sensitivity results in stronger reactions to what would be considered noise in a general population (and increases the risk of noise annoyance), while hyperacusis evokes adverse reactions to common sounds not generally thought of as noise. From the participants’ stories, being annoyed by the noise from the motorway is an expression of being noise sensitive, while not being able to stand birdsong indicates hyperacusis.

Noise sensitivity is an individual variation in sensibility to sound that is partly genetic [59] and therefore defined as a personality trait [60]. There seems to be a variation within a lifespan, possibly also influenced by sound exposure or other experiences, indicating that the concept of noise sensitivity comprises a more changeable trait [61]. Of a general population, 20%–30% could be classified as noise-sensitive when assessed by self-reporting protocols [62,63]. There is indication of an association between noise sensitivity and illness. In a cross-sectional study, highly noise-sensitive people reported higher prevalence of a number of symptoms, especially within psychological/neurovegetative, cardiovascular and ear-related categories [64]. This group was also overrepresented when it came to medically recorded non-specific physical symptoms and prescribed medication. Moreover, in a Finnish twin cohort study, the risk of cardiovascular mortality in the period 1989–2003 was found to be higher among women that rated themselves noise-sensitive in 1988 than among other women [65]. Another longitudinal study showed that people suffering from depression became less noise-sensitive as they recovered, although they remained more sensitive than the rest of the population [66]. Complementary physiological measurements in that study revealed prolonged reset time after arousal due to sound exposure among those sensitive, indicating a biological mechanism. Recently published results show significant differences in heart rate change and heart rate variability between noise-sensitive people and others [67]. Based on this, those authors suggested that noise sensitivity may be explained by a hypoactive parasympathetic response and a hyperactive and sustained sympathetic response, due to an uncoupling of the autonomic nervous system and the amygdala-prefrontal circuits that interpret stressful stimuli and enact the appropriate stress response. Whether noise sensitivity is the driver, or whether self-reported measurements of sensitivity indicate other underlying causes is not clear. It has been suggested that there is not a straight connection from noise sensitivity to ill health, or vice versa, but an individual vulnerability linked to both [68].

Hyperacusis as a category of sound susceptibility has previously mainly been reserved for consequences of e.g., migraine and tinnitus, and has hence been treated as an adverse effect of these illnesses (e.g., [69]) and not a subject for research in larger populations [70]. The definition has since been broadened, however, and hyperacusis is now considered an unusual intolerance to ordinary sounds more generally [71]. Few studies have assessed the prevalence in a common population, but that study found it to be approximately 10%. However, it is difficult to develop instruments for self-reporting that distinguish between increased noise sensitivity and hyperacusis. At the current state of knowledge, hyperacusis cannot be seen as a personal trait, but a state caused by illness or possible other stressors.

Among the participants in this study, some stated that they were noise-sensitive as a personality trait and that this noise sensitivity also had an impact on their behaviour in the rehabilitation garden. It could be hypothesised based on the literature that the prevalence of noise sensitivity is greater among people diagnosed with exhaustion disorder than in the general population, but this remains to be confirmed. What can be learned from this study, however, is that an increased susceptibility to noise and to sounds that are commonly not considered noise is customary among the target group. The descriptions we were given indicated that the stress the participants had been exposed to could place them in a state where it was not possible to process audio stimuli. Some of the participants chose a coping strategy whereby they shut down completely and stopped listening. Others tried to avoid noise as much as they could. Gradually, as they recovered in therapy, they were able to get accustomed to sounds again, although technological sounds were still problematic for some and an increase in sound awareness could lead to lowered noise tolerability. It is clear that the soundscape in such a process is of great importance.

### 4.3. Design Considerations

Our findings highlight several aspects that can be used to inform future design of environments for NBR treatment. Some of the findings can be directly related to design, such as sounds from a water feature (e.g., a ripple in a pond), or walking material (e.g., gravel or wood). Other things are indirectly related to design, such as sounds from twittering birds. There are also design measures that can be taken in order to avoid unwanted sounds, in the present case most typically the sound of the motorway and the sound of (certain) social activity.

From the survey material, we observed a general preference especially for sounds of nature, including the rustling of leaves, the rippling of water and the sound of birds. Sounds that were categorised as human also appeared to have a positive effect, such as the sound of one’s own footsteps (on wood or gravel), or the sound of the therapist’s voice. In designing rehabilitation gardens, the conditions for such wanted sounds could be accommodated.

Water features of different kinds have the potential to produce a range of different sounds. The possible variations in sonic character include rhythm, intensity and timbre, and can be considered in the design. The character of the sound influences appreciation as well as possibilities for masking effects [15,72]. The character may also have an influence on restorative qualities. One of the pioneers in soundscape research, suggests [2], for instance, that there is a connection between the rhythmic qualities of ocean waves and the soothing character that many people experience at the sea. That author argued that the average rhythm of waves corresponds to that of our breathing at rest. We have found no studies concerning rhythmic qualities of ocean waves, but it has been shown that the pace in music can influence the speed of everyday activities [73,74].

The materials on paths and other surfaces produce different sounds when walked upon. This can be used as a design feature. In our study, the sound of wood in particular was mentioned as a positive feature. Participants described how the sound that wood produced when walked upon encouraged them to move more slowly—thus presumably also reducing the activation level. In another context, it has been shown that the impact from noise can be reduced when there is sound from walking material [14]. In our study, a gravel path was used as a warning for approaching people. A similar function can be found in some wooden walkways from edo-era Japan, where a nightingale floor, *uguisubari*, is constructed in such a way that it creaks when walked upon—thus warning inhabitants of potential enemies approaching.

The rustling of vegetation is dependent on wind to reach the leaves to produce the effect. The mutual relationship between sound and wind can be taken into consideration with the choice of strategic windy position for plants—such as topographically higher positions, in open areas, or in corners of (tall) buildings. In this way, the vegetation may have a dual effect of reducing wind disturbance and producing pleasant sounds. Certain species are known to make more sounds than others in response to wind; the poplar genus (including aspen) and bamboo being examples of good “rustlers”. In China, it is commonly known that bamboo constitutes a good way of inviting the wind into a garden (along with maple and pine), and also of enhancing the sound of rain (along with plantain and lotus) [75].

In our study, birds were mentioned as a general category of (almost exclusively) pleasant sounds. There were no mentions of relative preference between individual species. However it has previously been found that, while birds are generally perceived as being a positive category of sound, certain species may have a negative effect. In a previous interview study on bird sound and restoration [76], it was found, for instance, that magpie, owls and crows could have negative associations for some people in terms of restoration. In a study concerning attitudes to different animals in Norway [77], it was reported that some citizens experienced problems with pigeons and seagulls, but that the general attitude towards both species was neutral. The combination (or lack of combination) of species may also have an effect—in a study investigating bird sound and environmental preference in different urban contexts [78], it was shown that increased birdsong diversity could have a positive effect on ratings. Considering the positive mentions of a variety of natural sounds in the present study, such diversity could be beneficial also in restorative contexts.

Birds can be attracted through creation of suitable biotopes [13]. Birds are generally attracted by basic affordances such as access to food (e.g., berries and insects), protection (e.g., shrubs, trees or birdhouses) and water [79,80]. Furthermore, songbirds are sensitive to vegetation structure and are attracted by plantings that are varied, dense and include different canopy layers. The age of vegetation may contribute as well—in forest stands, bird species diversity has been found to correlate with stand maturity [81,82], a factor which has also been linked to restorative qualities [83]. Water features can be designed with consideration for birds, so that there are shallower parts and components for birds to stand on, such as stones [84]. It is also possible to design specifically to attract (or discourage) certain species of birds through consideration of individual preferences [79]. In order to emphasise the relationship between biotopes and the sounds that are likely to be produced there—such as birds or any other animals that may have restorative potential [85]—the term sonotope [17] can be used.

A predominant approach to treatment of sound in environmental planning and design situations to date has been defensive strategies (noise management) [8,12]. In defensive strategies, the focus is directed towards sound pressure levels and protection from unwanted sounds. The present study confirmed the importance of considering noise; noise from the nearby motorway was perceived as a disturbing feature by several participants. Disturbance from such unwanted nearby activities, as was shown in our study, can be avoided through design considerations. In strategic locations, where there is sufficient distance from the unwanted sound, or in shelter behind buildings, the impact can be reduced. However, defensive strategies are not sufficient—the study also highlighted that the soundscape holds many other important qualities that should be given consideration in design of nature-based rehabilitation environments—not least because positive sonic experiences can be used to shift the focus from noise through masking [9,72,86].

In the present study, several references were made to silence as a positive quality—seemingly in line with the noise treatment strategy. However, the use of silence was never incorporated to refer to an acoustically measurable silence, such as the complete absence of sound. Silence was used, instead, to emphasise a tranquil state which included, most typically, presence of natural sounds. Traditional noise abatement should therefore be complemented with an experience perspective to form a holistic soundscape approach in which qualities such as those indicated in the study are given due recognition.

## 5. Conclusions

To date, few studies have considered the role of soundscapes in the context of nature-based rehabilitation (NBR). The interview material studied here was not collected with the intention of focusing on sound, yet sound was mentioned spontaneously by around half the interviewees. Three sonic themes were examined in the study; natural, technological and human sounds. In addition to the main themes, three cross-theme dimensions emerged; sound in relation to overall perception; sound in relation to garden usage; and increased susceptibility. Given the qualitative nature of our study, the results should not be regarded as generalizable, but rather transferable. For instance, the results could be used to inform other garden therapy situations or similar contexts.

The study showed that sound influenced experience and behaviour in the garden, and that it can play several different roles in NBR—with positive and negative effects on the rehabilitation process. Sounds of nature were almost exclusively considered to be a positive element in the garden—the descriptions of nature sounds indicated that sound and other sensory experiences play an important role in ‘soft fascination’ and natural sounds also seemed prone to stimulate memories. Technological sounds were exclusively considered to be a negative element—yet the perceived disturbance from the nearby motorway in the garden varied between participants, and seemed to be set in relation to other environments to which the patient was accustomed.

Human sounds emerged as the most complex of the three themes that were studied. The preference seemed to vary between different individuals, moods and treatment phases. For several participants, it was crucial to be able to avoid social interaction with other people—especially in early phases of the treatments—which relates to the notion of ‘social quietness’. On the other hand, presence of human sounds that did not require interaction (for instance the sound of someone working in the distance, or the sound of a therapist’s voice) seemed to be much less disturbing, and could even be reassuring—for instance, several participants referred to the therapists’ voices in a positive manner.

The study produced several findings of value for design and maintenance regarding sounds in rehabilitation gardens. Some of these are general, while others—especially those concerning human sounds—are more complex. Suggested recommendations on how to implement the findings in design could be to make a garden that is varied and diverse as regards e.g., water features, vegetation and size of the garden. Variation would allow each participant to seek out their own favourite soundscape, based on aspects such as personality, mood and treatment phase, and meet the needs of different stages of sound sensibility. The role of variation also tallies with previous findings in the same rehabilitation garden that self-chosen places are an important aspect to consider in the garden. In our study, we saw that the actual act in which places were chosen, or avoided (based on the sounds), could take several forms, some of which were creative and innovative.

This study contributed with a perspective that, while studied in other contexts, has hitherto been given relatively little attention in NBR. Our findings suggest that sound should be taken into account when considering future design and management of rehabilitation gardens. It should be stressed, however, that environmental sounds constitute but one aspect of the environmental experience. As a consequence, not only sound needs to be given increased attention, but also the interplay between sound and other cues, like visual information. Our study indicated that such relationships could be of importance. Furthermore, and finally, we believe that an increased knowledge on sound in NBR could be a useful means to inform and further develop existing theories and models on nature-based rehabilitation.

## Appendix A

Thesaurus used for retrieving units of meaning from the transcribed interviews. The English translations give an indication of the words that were used, but not the exact meanings, as the words were often onomatopoeic, making them less suitable for direct translation.

**Table A1:** Thesaurus of keywords.

Swedish Thesaurus	English Approximation
ljud * (ljud, ljudet, billjud, ljudkänslig, motorvägsljudet, trafikljud)	sound * (sound, the sound, car sound, sound sensitivity, motorway sounds, traffic sounds)
tyst * (tyst, tystnad)	quiet * (quiet, quietness)
hör * (höra, hörs, hört, hörsel)	hear * (hear, heard, hearing)
bull * (buller, bullrig)	noise * (noise, noisy)
prat * (prat, pratar)	talk * (talk, talking)
rassl * (prasslande, prassla, rassla, rasslande)	clutter * (rustle, rustling, rattle, rattling, clutter, cluttering)
porl * (porla, porlande)	trickle * (tricking, rippling)
kluck * (klucka)	lap * (lapping)
brus * (brusa)	rush * (rushing)
rinn * (rinna)	flow * (flowing)
sus * (susa, susande)	rustle * (rustling)
brum * (brummande)	throb * (throbbing)
surr * (surra, surrande)	buzz * (buzzing)
knast * (knastra, knastrat)	crunch * (crunching)

* Indicate keywords used to search the transcribed interviews. Examples of words that the search returned are indicated in brackets.
